# 9-Amino­acridinium bis­(pyridine-2,6-dicarboxyl­ato-κ^3^ *O* ^2^,*N*,*O* ^6^)ferrate(III) tetra­hydrate

**DOI:** 10.1107/S1600536812020247

**Published:** 2012-05-12

**Authors:** Masoud Mirzaei, Hossein Eshtiagh-Hosseini, Ehsan Eydizadeh, Zakieh Yousefi, Krešimir Molčanov

**Affiliations:** a Department of Chemistry, Ferdowsi University of Mashhad, 917791436 Mashhad, Iran; b Laboratory of Chemical Crystallography and Biocrystallography, Department of Physical Chemistry, Rudjer Bošković Institute, Bijenička 54, HR-10000, Zagreb, Croatia

## Abstract

The asymmetric unit of the title compound, (C_13_H_11_N_2_)[Fe(C_7_H_3_NO_4_)_2_]·4H_2_O, contains a 9-amino­acridinium cation, one anionic complex and four uncoordinated water mol­ecules. In the anionic complex, the Fe^III^ ion is six-coordinated by two almost perpendicular [dihedral angle = 88.78 (7)°] pyridine-2,6-dicarboxyl­ate ligands in a distorted octa­hedral geometry. In the crystal, anions are connected into chains along [10-1] by weak C—H⋯O inter­actions, which create ten-membered hydrogen-bonded *R*_2_^2^(10) rings. These chains are linked by three-membered water clusters. The final three-dimensional network is constructed by numerous inter­molecular O—H⋯O and N—H⋯O inter­actions.

## Related literature

For background to supra­molecular chemistry, see: Lehn (2002 ▶). For functionalized materials, see: Moulton & Zaworotko (2001 ▶). For a brief reviews on the pyridine­dicarboxyl­ate family of ligands, see: Mirzaei *et al.* (2011 ▶); Axelrod *et al.* (2000 ▶). For the role of water clusters, see: Aghabozorg *et al.* (2010 ▶). For related structures: Aghabozorg *et al.* (2008 ▶); Eshtiagh-Hosseini *et al.* (2010*a* ▶,*b* ▶, 2011*a* ▶,*b* ▶).
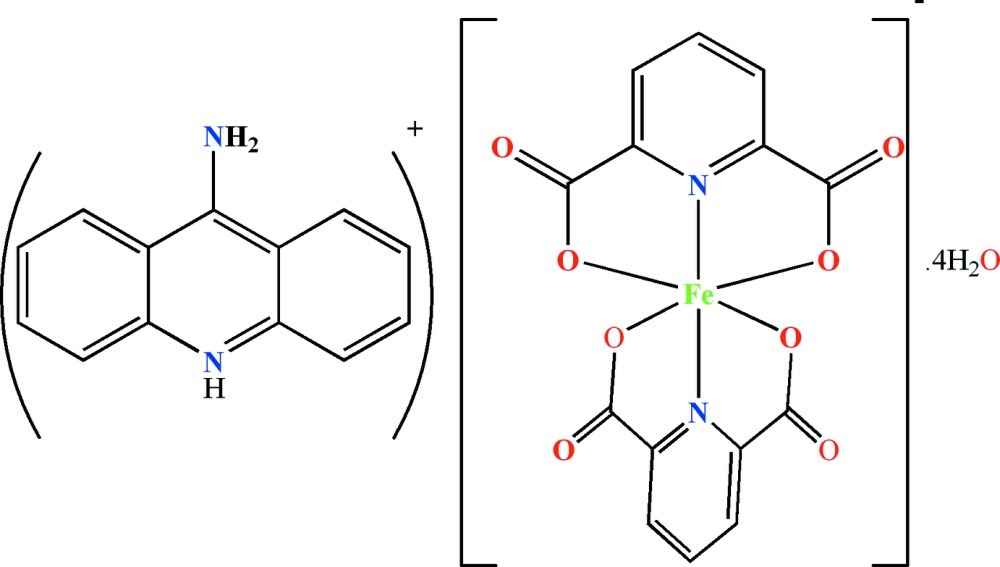


## Experimental

### 

#### Crystal data

(C_13_H_11_N_2_)[Fe(C_7_H_3_NO_4_)_2_]·4H_2_O
*M* *_r_* = 653.36
Monoclinic, 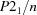

*a* = 9.6130 (1) Å
*b* = 18.9256 (2) Å
*c* = 15.9563 (2) Å
β = 96.037 (1)°
*V* = 2886.86 (6) Å^3^
*Z* = 4
Cu *K*α radiation
μ = 4.77 mm^−1^
*T* = 293 K
0.2 × 0.15 × 0.1 mm

#### Data collection

Agilent Xcalibur Ruby Nova diffractometer
Absorption correction: multi-scan (*CrysAlis PRO*; Agilent, 2011 ▶) *T*_min_ = 0.602, *T*_max_ = 1
15252 measured reflections
5940 independent reflections
5140 reflections with *I* > 2σ(*I*)
*R*_int_ = 0.020

#### Refinement

*R*[*F* ^2^ > 2σ(*F* ^2^)] = 0.059
*wR*(*F* ^2^) = 0.178
*S* = 1.05
5940 reflections
430 parameters
14 restraints
H atoms treated by a mixture of independent and constrained refinement
Δρ_max_ = 0.86 e Å^−3^
Δρ_min_ = −0.33 e Å^−3^

### 

Data collection: *CrysAlis PRO* (Agilent, 2011 ▶); cell refinement: *CrysAlis PRO*; data reduction: *CrysAlis PRO*; program(s) used to solve structure: *SHELXS97* (Sheldrick, 2008 ▶); program(s) used to refine structure: *SHELXL97* (Sheldrick, 2008 ▶); molecular graphics: *ORTEP-3 for Windows* (Farrugia, 1997 ▶); software used to prepare material for publication: *WinGX* (Farrugia, 1999 ▶).

## Supplementary Material

Crystal structure: contains datablock(s) global, I. DOI: 10.1107/S1600536812020247/bq2350sup1.cif

Structure factors: contains datablock(s) I. DOI: 10.1107/S1600536812020247/bq2350Isup2.hkl

Additional supplementary materials:  crystallographic information; 3D view; checkCIF report

## Figures and Tables

**Table 1:** Hydrogen-bond geometry (Å, °)

*D*—H⋯*A*	*D*—H	H⋯*A*	*D*⋯*A*	*D*—H⋯*A*
N3—H3*A*⋯O3	0.86	2.42	3.038 (4)	130
N3—H3*A*⋯O11	0.86	2.30	3.008 (4)	139
N4—H4*A*⋯O12^i^	0.86	2.03	2.822 (5)	152
N4—H4*B*⋯O5^ii^	0.86	2.39	3.115 (4)	142
O9—H9*A*⋯O6	0.93 (4)	1.81 (5)	2.726 (4)	165 (5)
O9—H9*B*⋯O11^iii^	0.92 (2)	1.85 (2)	2.766 (4)	170 (5)
O10—H10*A*⋯O9^iv^	0.97 (5)	1.81 (5)	2.750 (5)	164 (5)
O10—H10*B*⋯O1	0.97 (5)	1.90 (5)	2.859 (5)	176 (11)
O11—H11*A*⋯O4	0.95 (4)	1.78 (4)	2.715 (4)	165 (3)
O11—H11*B*⋯O8^v^	0.93 (4)	1.97 (2)	2.865 (4)	163 (4)
O12—H12*A*⋯O10^vi^	0.96 (6)	1.96 (7)	2.827 (5)	149 (7)
O12—H12*B*⋯O8	0.95 (11)	2.06 (8)	2.874 (4)	142 (10)
C4—H4⋯O6^vii^	0.93	2.41	3.334 (4)	171
C9—H9⋯O4^viii^	0.93	2.33	3.257 (4)	171
C16—H16⋯O12^v^	0.93	2.59	3.426 (6)	150
C17—H17⋯O2^ix^	0.93	2.54	3.329 (5)	143

Symmetry codes: (i) 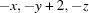; (ii) 
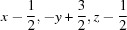
; (iii) 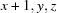; (iv) 
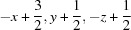
; (v) 
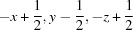
; (vi) 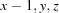; (vii) 
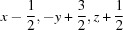
; (viii) 
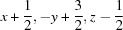
; (ix) 
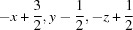
.

**Fig. 1.**
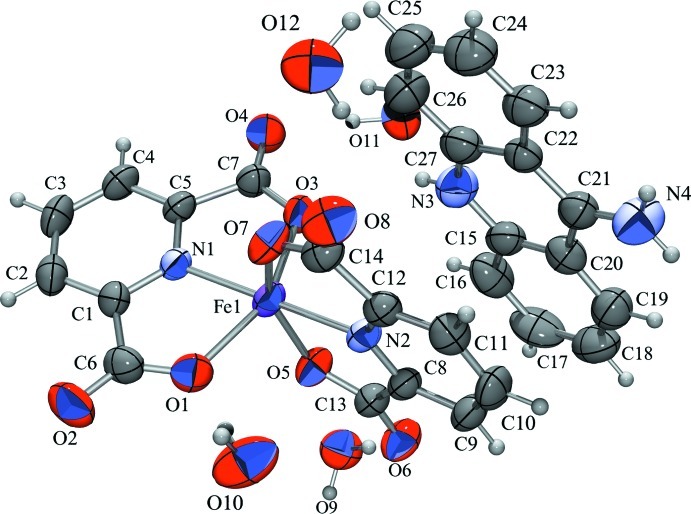
An ORTEP view of the title compound with numbering non-hydrogen atoms with probability 50%.

**Fig. 2.**
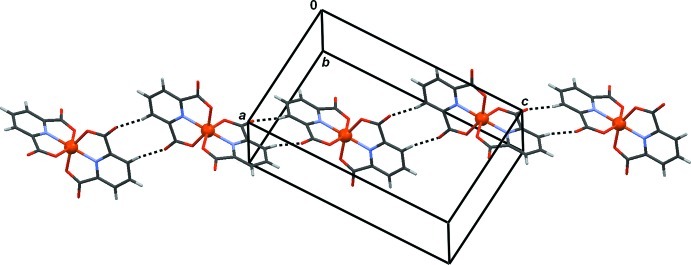
A representation of 1-D chains formed by anionic complexes with considering related synthons.

**Fig. 3.**
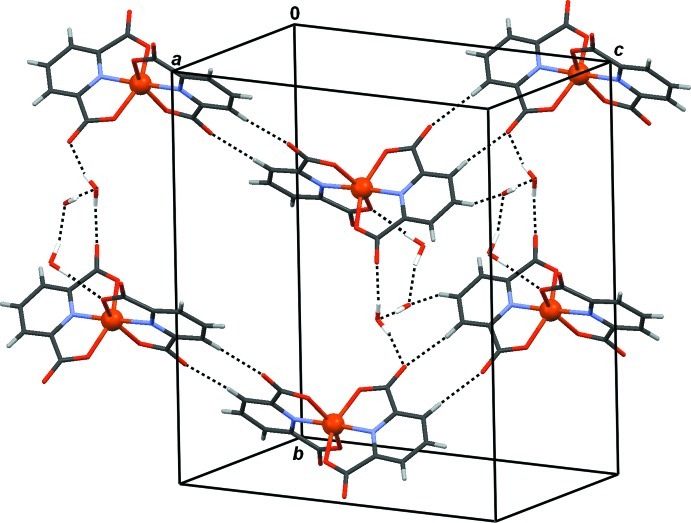
The role of water clusters in connection of 1-D chains.

**Fig. 4.**
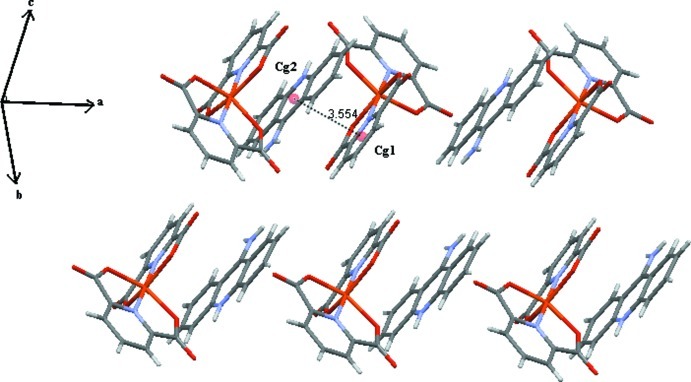
Packing diagram of the title compounds with considering π—π stacking between cationic and anionic parts (water molecules have been omitted; Cg1: N2, C, C9, C10, C11, C12 and Cg2: N3, C15, C20, C21, C22, C27).

## supplementary crystallographic information

### Comment

Supramolecular chemistry, the knowledge of weak intermolecular interactions, has been attracting attention of basic sciences researchers and crystallographers (Lehn, 2002).

The functionalized materials such as dicarboxylic acids, amines and amides are important in this area (Moulton & Zaworotko, 2001).

Since 2008, we have focused on polycarboxylic acid complexes with transition metal ions along with N–, O–, and S– donor ligands for better clarification of non-covalent and coordination interactions of these ligands in natural human system, food chemistry, medicine *etc* (Mirzaei *et al.*, 2011*a*; Axelrod *et al.*, 2000).

Among them, pyridine-2,6-dicarboxylic acid with two carboxylic groups and a heteroaromatic ring has capability of participating in intermolecular interactions. Also, H_2_pydc and its mono- or doubly protonated form with high symmetry and four electron donating oxygen atoms and one nitrogen atom can be applied as a multidendate ligand in coordination compounds which can possess various coordination modes (Aghabozorg *et al.*, 2008; Mirzaei *et al.*, 2011). The most common coordination mode for (pydc)^2-^ is tridentate: two (pydc)^2-^ are coordinated to metal and induce octahedral coordination environment to the metal ion (Eshtiagh-Hosseini *et al.*, 2010*b*). In this case, a counter ion is required for compensation of charge, for example, (Hbmmpa)[Fe(pydc)_2_].(EtOH)_0.8_(H_2_O)_0.2_ (bmmpa is short for 5-bromo-6-methyl-2-morpholinepyrimidine-4-amine, Eshtiagh-Hosseini *et al.*, 2010*a*) and (H2-apym)[Fe(pydc)_2_].3H_2_O (2-apym is abbreviation of 2-aminopyrimidine, Eshtiagh-Hosseini *et al.* 2011*a*).

In continuation of our studies, we have synthesized and structurally characterized a new crystalline coordination compound, (H9-Acr)[Fe(pydc)_2_].4H_2_O.

Fe^III^ has been coordinated by two almost perpendicular tridentate ligands (dihedral angle 88.78 (7)° ) with distorted octahedral geometry; a protonated 9-Acr moiety is present as a cation (Fig. 1).

In crystalline network, anionic complexes are connected to each other by C—H···O (〈D—H···A: 170.93°) interactions which can create a supramolecular synthon with graph set *R*^2^_2_(10) in [101] direction (Fig. 2). These chains are attached to each other by three membered water cluster (Fig.3). In spite of the most recently observation which π-π interactions created between acridine moieties (Eshtiagh-Hosseini *et al.*, 2011*b*), no π–π interaction between H9-Acr moieties is observed. Instead, such an interaction can be observed between anionic and cationic parts as seen in Fig. 4 that may be important in the formation of the ultimate network.

### Experimental

To an aqeous solution (5 ml) of pydcH_2_ (0.034 g, 0.2 mmol), 9-Acr (0.020 g, 0.1 mmol) in methanol (10 ml) solution was added dropwise following which a solution of FeCl_3_.6H_2_O (0.027 g, 0.1 mmol) in water (2 ml) was added and the resultant solution was heated and stirred for 3 hrs at 60 °C. Yellow crystals were obtained by slow evaporation of the solvent at room temperature after a week.

### Refinement

A full-matrix least-squares refinement implemented in the *SHELXL97* (Sheldrick, 2008) was used. All non-H atoms were refined anisotropically. The H atoms were positioned geometrically and allowed to ride on their parent atoms, with C—H = 0.93 Å and 0.97 Å for C and 0.86 Å for N atom and *U_iso_*(H) = 1.2 *U_eq_*(C,*N*). The H atoms of water were located in difference map and refined with the following restraints: O—H = 0.95 (2) Å and H···H = 1.50 (4) Å (total of 14 restraints were used).

### Crystal data

**Table:**

(C_13_H_11_N_2_)[Fe(C_7_H_3_NO_4_)_2_]·4H_2_O	*F*(000) = 1348
*M**_r_* = 653.36	*D*_x_ = 1.503 Mg m^−^^3^
Monoclinic, *P*2_1_/*n*	Cu *K*α radiation, λ = 1.54184 Å
Hall symbol: -P 2yn	Cell parameters from 7426 reflections
*a* = 9.6130 (1) Å	θ = 3.6–75.8°
*b* = 18.9256 (2) Å	µ = 4.77 mm^−^^1^
*c* = 15.9563 (2) Å	*T* = 293 K
β = 96.037 (1)°	Prism, yellow
*V* = 2886.86 (6) Å^3^	0.2 × 0.15 × 0.1 mm
*Z* = 4	

### Data collection

**Table:**

Agilent Xcalibur Ruby Nova diffractometer	5140 reflections with *I* > 2σ(*I*)
ω scans	*R*_int_ = 0.020
Absorption correction: multi-scan (*CrysAlis PRO*; Agilent, 2011)	θ_max_ = 76.0°, θ_min_ = 3.6°
*T*_min_ = 0.602, *T*_max_ = 1	*h* = −11→12
15252 measured reflections	*k* = −23→20
5940 independent reflections	*l* = −19→17

### Refinement

**Table:**

Refinement on *F*^2^	H atoms treated by a mixture of independent and constrained refinement
Least-squares matrix: full	*w* = 1/[σ^2^(*F*_o_^2^) + (0.1074*P*)^2^ + 1.5769*P*] where *P* = (*F*_o_^2^ + 2*F*_c_^2^)/3
*R*[*F*^2^ > 2σ(*F*^2^)] = 0.059	(Δ/σ)_max_ = 0.001
*wR*(*F*^2^) = 0.178	Δρ_max_ = 0.86 e Å^−^^3^
*S* = 1.05	Δρ_min_ = −0.33 e Å^−^^3^
5940 reflections	Extinction correction: *SHELXS97* (Sheldrick, 2008), Fc^*^=kFc[1+0.001xFc^2^λ^3^/sin(2θ)]^-1/4^
430 parameters	Extinction coefficient: 0.0010 (3)
14 restraints	

### Special details

**Table:**

Geometry. All esds (except the esd in the dihedral angle between two l.s. planes) are estimated using the full covariance matrix. The cell esds are taken into account individually in the estimation of esds in distances, angles and torsion angles; correlations between esds in cell parameters are only used when they are defined by crystal symmetry. An approximate (isotropic) treatment of cell esds is used for estimating esds involving l.s. planes.

### Fractional atomic coordinates and isotropic or equivalent isotropic displacement parameters (Å^2^)

**Table:**

	*x*	*y*	*z*	*U*_iso_*/*U*_eq_	
Fe1	0.52343 (5)	0.88273 (2)	0.30488 (3)	0.04513 (18)	
O1	0.6777 (3)	0.95237 (13)	0.33779 (15)	0.0614 (6)	
O2	0.7959 (3)	1.01376 (15)	0.4417 (2)	0.0796 (8)	
O3	0.3790 (2)	0.81178 (12)	0.33429 (13)	0.0532 (5)	
O4	0.2764 (3)	0.75917 (14)	0.43703 (16)	0.0697 (7)	
O5	0.6572 (2)	0.80542 (12)	0.27746 (13)	0.0535 (5)	
O6	0.7560 (3)	0.74841 (14)	0.17669 (16)	0.0657 (6)	
O7	0.3804 (3)	0.95880 (13)	0.26910 (15)	0.0611 (6)	
O8	0.2689 (3)	1.01957 (14)	0.1621 (2)	0.0754 (8)	
N1	0.5332 (2)	0.88765 (12)	0.43414 (15)	0.0417 (5)	
N2	0.5108 (3)	0.88283 (12)	0.17512 (15)	0.0418 (5)	
C1	0.6238 (3)	0.93173 (15)	0.47541 (18)	0.0451 (6)	
C2	0.6292 (4)	0.93776 (18)	0.5628 (2)	0.0550 (7)	
H2	0.6926	0.968	0.5925	0.066*	
C3	0.5380 (4)	0.89773 (19)	0.6036 (2)	0.0592 (8)	
H3	0.5386	0.9016	0.6618	0.071*	
C4	0.4454 (4)	0.85176 (18)	0.55960 (19)	0.0530 (7)	
H4	0.3846	0.8242	0.5874	0.064*	
C5	0.4457 (3)	0.84784 (15)	0.47327 (18)	0.0430 (6)	
C6	0.7092 (3)	0.97043 (16)	0.4157 (2)	0.0550 (7)	
C7	0.3576 (3)	0.80208 (16)	0.41175 (19)	0.0479 (6)	
C8	0.5914 (3)	0.83863 (15)	0.13740 (17)	0.0424 (6)	
C9	0.5902 (3)	0.83856 (18)	0.0508 (2)	0.0534 (7)	
H9	0.6478	0.8084	0.0241	0.064*	
C10	0.5001 (4)	0.88496 (19)	0.0052 (2)	0.0594 (8)	
H10	0.496	0.8858	−0.0533	0.071*	
C11	0.4157 (3)	0.93029 (17)	0.0460 (2)	0.0536 (7)	
H11	0.3546	0.9613	0.0156	0.064*	
C12	0.4250 (3)	0.92806 (14)	0.13266 (19)	0.0443 (6)	
C13	0.6774 (3)	0.79258 (16)	0.20043 (19)	0.0467 (6)	
C14	0.3490 (3)	0.97350 (16)	0.1909 (2)	0.0529 (7)	
N3	0.2278 (3)	0.76041 (19)	0.1687 (2)	0.0685 (8)	
H3A	0.2451	0.7491	0.2209	0.082*	
N4	0.1409 (4)	0.80668 (19)	−0.0810 (2)	0.0756 (9)	
H4A	0.0776	0.8374	−0.097	0.091*	
H4B	0.1847	0.7852	−0.1178	0.091*	
C15	0.3006 (3)	0.72748 (18)	0.1104 (2)	0.0562 (7)	
C16	0.4026 (4)	0.6782 (2)	0.1433 (3)	0.0755 (11)	
H16	0.417	0.6688	0.2007	0.091*	
C17	0.4812 (4)	0.6441 (2)	0.0865 (4)	0.0830 (14)	
H17	0.5494	0.6115	0.106	0.1*	
C18	0.4577 (4)	0.6589 (2)	0.0009 (3)	0.0800 (12)	
H18	0.5114	0.6364	−0.0364	0.096*	
C19	0.3580 (4)	0.7053 (2)	−0.0290 (3)	0.0686 (9)	
H19	0.3424	0.7135	−0.0867	0.082*	
C20	0.2791 (3)	0.74077 (17)	0.0250 (2)	0.0549 (7)	
C21	0.1699 (3)	0.79300 (18)	−0.0026 (2)	0.0565 (8)	
C22	0.0989 (3)	0.82788 (15)	0.05985 (19)	0.0468 (6)	
C23	−0.0044 (4)	0.88057 (18)	0.0422 (3)	0.0637 (9)	
H23	−0.0291	0.8932	−0.0138	0.076*	
C24	−0.0686 (5)	0.9133 (2)	0.1019 (3)	0.0802 (12)	
H24	−0.1351	0.948	0.0873	0.096*	
C25	−0.0347 (4)	0.8947 (3)	0.1863 (3)	0.0823 (13)	
H25	−0.0796	0.9171	0.2279	0.099*	
C26	0.0625 (5)	0.8445 (3)	0.2084 (3)	0.0775 (11)	
H26	0.0843	0.833	0.265	0.093*	
C27	0.1307 (4)	0.8097 (2)	0.1468 (3)	0.0618 (8)	
O9	0.8858 (3)	0.66602 (17)	0.30146 (19)	0.0750 (7)	
H9A	0.841 (5)	0.687 (3)	0.253 (2)	0.115 (19)*	
H9B	0.982 (2)	0.671 (3)	0.302 (3)	0.089 (15)*	
O10	0.7306 (4)	1.03358 (19)	0.1934 (3)	0.0988 (11)	
H10A	0.675 (6)	1.076 (2)	0.199 (4)	0.14 (2)*	
H10B	0.710 (9)	1.008 (3)	0.243 (3)	0.21 (4)*	
O11	0.1750 (3)	0.66734 (15)	0.31482 (18)	0.0691 (7)	
H11A	0.210 (5)	0.693 (2)	0.364 (2)	0.097 (16)*	
H11B	0.206 (4)	0.6226 (12)	0.331 (3)	0.069 (12)*	
O12	0.0023 (3)	1.08479 (18)	0.1758 (2)	0.0844 (8)	
H12A	−0.090 (5)	1.067 (7)	0.160 (5)	1.1 (4)*	
H12B	0.063 (7)	1.052 (8)	0.153 (10)	0.53 (13)*	

### Atomic displacement parameters (Å^2^)

**Table:**

	*U*^11^	*U*^22^	*U*^33^	*U*^12^	*U*^13^	*U*^23^
Fe1	0.0566 (3)	0.0466 (3)	0.0345 (3)	−0.00371 (19)	0.01561 (18)	−0.00198 (16)
O1	0.0685 (14)	0.0605 (13)	0.0584 (13)	−0.0183 (11)	0.0217 (11)	0.0015 (11)
O2	0.0737 (16)	0.0708 (16)	0.094 (2)	−0.0321 (14)	0.0055 (14)	−0.0027 (15)
O3	0.0645 (13)	0.0585 (12)	0.0382 (10)	−0.0174 (10)	0.0122 (9)	−0.0063 (9)
O4	0.0761 (15)	0.0758 (16)	0.0608 (14)	−0.0338 (13)	0.0245 (12)	−0.0043 (12)
O5	0.0655 (13)	0.0558 (12)	0.0406 (11)	0.0111 (10)	0.0117 (9)	0.0035 (9)
O6	0.0697 (14)	0.0677 (15)	0.0609 (14)	0.0242 (12)	0.0130 (11)	−0.0066 (11)
O7	0.0762 (15)	0.0572 (13)	0.0532 (13)	0.0114 (11)	0.0221 (11)	−0.0085 (10)
O8	0.0786 (17)	0.0567 (14)	0.092 (2)	0.0225 (13)	0.0144 (14)	0.0045 (13)
N1	0.0460 (12)	0.0426 (12)	0.0380 (12)	−0.0022 (9)	0.0118 (9)	−0.0038 (9)
N2	0.0484 (12)	0.0404 (12)	0.0387 (12)	−0.0015 (9)	0.0141 (9)	0.0007 (9)
C1	0.0482 (14)	0.0424 (13)	0.0450 (14)	−0.0003 (11)	0.0061 (11)	−0.0074 (11)
C2	0.0610 (18)	0.0549 (17)	0.0475 (16)	0.0039 (14)	−0.0014 (13)	−0.0139 (13)
C3	0.076 (2)	0.0651 (19)	0.0362 (15)	0.0081 (17)	0.0064 (14)	−0.0077 (13)
C4	0.0615 (17)	0.0594 (17)	0.0407 (15)	0.0040 (14)	0.0176 (13)	0.0025 (13)
C5	0.0469 (14)	0.0454 (14)	0.0388 (13)	0.0007 (11)	0.0142 (11)	−0.0002 (11)
C6	0.0542 (16)	0.0437 (15)	0.068 (2)	−0.0072 (13)	0.0114 (14)	−0.0008 (14)
C7	0.0514 (15)	0.0509 (15)	0.0436 (14)	−0.0088 (12)	0.0151 (12)	−0.0008 (12)
C8	0.0453 (13)	0.0434 (13)	0.0403 (14)	−0.0019 (11)	0.0129 (11)	−0.0031 (11)
C9	0.0601 (17)	0.0583 (17)	0.0446 (15)	−0.0004 (14)	0.0188 (13)	−0.0067 (13)
C10	0.073 (2)	0.073 (2)	0.0336 (15)	−0.0046 (16)	0.0093 (13)	0.0006 (13)
C11	0.0574 (17)	0.0527 (16)	0.0499 (16)	−0.0040 (13)	0.0031 (13)	0.0082 (13)
C12	0.0476 (14)	0.0382 (13)	0.0482 (15)	−0.0040 (11)	0.0103 (11)	0.0031 (11)
C13	0.0494 (14)	0.0479 (15)	0.0442 (15)	0.0023 (12)	0.0110 (11)	−0.0021 (12)
C14	0.0572 (17)	0.0412 (14)	0.0623 (19)	0.0028 (13)	0.0159 (14)	−0.0011 (13)
N3	0.0682 (18)	0.083 (2)	0.0547 (16)	0.0035 (16)	0.0074 (13)	0.0079 (15)
N4	0.081 (2)	0.075 (2)	0.072 (2)	0.0226 (17)	0.0148 (16)	0.0112 (17)
C15	0.0481 (15)	0.0581 (17)	0.0643 (19)	−0.0061 (14)	0.0142 (14)	−0.0041 (15)
C16	0.073 (2)	0.083 (3)	0.068 (2)	−0.009 (2)	−0.0075 (19)	0.020 (2)
C17	0.0507 (19)	0.064 (2)	0.132 (4)	0.0162 (17)	−0.001 (2)	0.011 (2)
C18	0.066 (2)	0.078 (3)	0.100 (3)	0.000 (2)	0.028 (2)	−0.016 (2)
C19	0.074 (2)	0.070 (2)	0.063 (2)	−0.0053 (18)	0.0125 (17)	−0.0034 (17)
C20	0.0540 (16)	0.0473 (15)	0.0628 (19)	−0.0045 (13)	0.0028 (14)	0.0047 (13)
C21	0.0541 (16)	0.0565 (17)	0.0598 (19)	−0.0091 (14)	0.0106 (14)	−0.0034 (14)
C22	0.0412 (13)	0.0451 (14)	0.0540 (16)	−0.0046 (11)	0.0049 (11)	−0.0008 (12)
C23	0.0570 (18)	0.0532 (18)	0.082 (3)	0.0021 (14)	0.0099 (17)	−0.0086 (16)
C24	0.072 (2)	0.070 (2)	0.100 (3)	0.010 (2)	0.015 (2)	−0.007 (2)
C25	0.075 (3)	0.083 (3)	0.093 (3)	0.002 (2)	0.027 (2)	−0.021 (2)
C26	0.075 (2)	0.092 (3)	0.068 (2)	−0.006 (2)	0.0185 (19)	−0.009 (2)
C27	0.0504 (17)	0.0624 (19)	0.074 (2)	−0.0068 (15)	0.0104 (15)	−0.0053 (17)
O9	0.0724 (17)	0.0870 (19)	0.0651 (16)	−0.0028 (15)	0.0058 (13)	0.0152 (14)
O10	0.120 (3)	0.077 (2)	0.109 (3)	0.0106 (19)	0.056 (2)	0.0198 (19)
O11	0.0763 (16)	0.0622 (15)	0.0669 (16)	−0.0011 (13)	−0.0014 (13)	0.0011 (12)
O12	0.0805 (18)	0.0745 (18)	0.098 (2)	0.0113 (15)	0.0102 (16)	0.0118 (16)

### Geometric parameters (Å, º)

**Table:**

Fe1—O1	2.012 (2)	N3—C27	1.341 (5)
Fe1—O3	2.022 (2)	N3—C15	1.371 (5)
Fe1—O5	2.026 (2)	N3—H3A	0.86
Fe1—O7	2.031 (2)	N4—C21	1.280 (5)
Fe1—N1	2.057 (2)	N4—H4A	0.86
Fe1—N2	2.061 (2)	N4—H4B	0.86
O1—C6	1.294 (4)	C15—C20	1.379 (5)
O2—C6	1.211 (4)	C15—C16	1.414 (5)
O3—C7	1.287 (4)	C16—C17	1.397 (7)
O4—C7	1.224 (4)	C16—H16	0.93
O5—C13	1.288 (4)	C17—C18	1.389 (7)
O6—C13	1.214 (4)	C17—H17	0.93
O7—C14	1.283 (4)	C18—C19	1.350 (6)
O8—C14	1.221 (4)	C18—H18	0.93
N1—C1	1.329 (4)	C19—C20	1.381 (5)
N1—C5	1.333 (4)	C19—H19	0.93
N2—C12	1.324 (4)	C20—C21	1.475 (5)
N2—C8	1.327 (4)	C21—C22	1.428 (5)
C1—C2	1.394 (4)	C22—C23	1.415 (5)
C1—C6	1.511 (4)	C22—C27	1.431 (5)
C2—C3	1.374 (5)	C23—C24	1.340 (6)
C2—H2	0.93	C23—H23	0.93
C3—C4	1.382 (5)	C24—C25	1.398 (7)
C3—H3	0.93	C24—H24	0.93
C4—C5	1.380 (4)	C25—C26	1.353 (7)
C4—H4	0.93	C25—H25	0.93
C5—C7	1.502 (4)	C26—C27	1.402 (6)
C8—C9	1.380 (4)	C26—H26	0.93
C8—C13	1.510 (4)	O9—H9A	0.928 (19)
C9—C10	1.385 (5)	O9—H9B	0.924 (19)
C9—H9	0.93	O10—H10A	0.97 (2)
C10—C11	1.388 (5)	O10—H10B	0.96 (2)
C10—H10	0.93	O11—H11A	0.946 (19)
C11—C12	1.377 (4)	O11—H11B	0.926 (18)
C11—H11	0.93	O12—H12A	0.96 (2)
C12—C14	1.510 (4)	O12—H12B	0.96 (2)
			
O1—Fe1—O3	151.61 (9)	N2—C12—C11	120.3 (3)
O1—Fe1—O5	93.57 (10)	N2—C12—C14	111.6 (3)
O3—Fe1—O5	92.15 (10)	C11—C12—C14	128.0 (3)
O1—Fe1—O7	93.89 (12)	O6—C13—O5	126.1 (3)
O3—Fe1—O7	94.29 (10)	O6—C13—C8	120.3 (3)
O5—Fe1—O7	151.37 (9)	O5—C13—C8	113.6 (2)
O1—Fe1—N1	75.75 (9)	O8—C14—O7	126.5 (3)
O3—Fe1—N1	75.96 (9)	O8—C14—C12	120.2 (3)
O5—Fe1—N1	106.63 (9)	O7—C14—C12	113.3 (3)
O7—Fe1—N1	102.00 (9)	C27—N3—C15	122.1 (3)
O1—Fe1—N2	103.03 (9)	C27—N3—H3A	119
O3—Fe1—N2	105.34 (9)	C15—N3—H3A	119
O5—Fe1—N2	75.84 (9)	C21—N4—H4A	120
O7—Fe1—N2	75.54 (9)	C21—N4—H4B	120
N1—Fe1—N2	177.24 (9)	H4A—N4—H4B	120
C6—O1—Fe1	121.0 (2)	N3—C15—C20	123.6 (3)
C7—O3—Fe1	120.05 (19)	N3—C15—C16	115.5 (4)
C13—O5—Fe1	120.35 (19)	C20—C15—C16	120.9 (3)
C14—O7—Fe1	120.66 (19)	C17—C16—C15	117.7 (4)
C1—N1—C5	122.4 (3)	C17—C16—H16	121.2
C1—N1—Fe1	118.93 (19)	C15—C16—H16	121.2
C5—N1—Fe1	118.67 (19)	C18—C17—C16	120.1 (4)
C12—N2—C8	122.5 (3)	C18—C17—H17	119.9
C12—N2—Fe1	118.83 (19)	C16—C17—H17	119.9
C8—N2—Fe1	118.65 (19)	C19—C18—C17	121.0 (4)
N1—C1—C2	120.0 (3)	C19—C18—H18	119.5
N1—C1—C6	111.3 (3)	C17—C18—H18	119.5
C2—C1—C6	128.7 (3)	C18—C19—C20	120.8 (4)
C3—C2—C1	118.1 (3)	C18—C19—H19	119.6
C3—C2—H2	120.9	C20—C19—H19	119.6
C1—C2—H2	120.9	C15—C20—C19	119.5 (3)
C2—C3—C4	121.0 (3)	C15—C20—C21	116.4 (3)
C2—C3—H3	119.5	C19—C20—C21	124.1 (3)
C4—C3—H3	119.5	N4—C21—C22	121.2 (3)
C5—C4—C3	118.2 (3)	N4—C21—C20	120.2 (3)
C5—C4—H4	120.9	C22—C21—C20	118.6 (3)
C3—C4—H4	120.9	C23—C22—C21	124.3 (3)
N1—C5—C4	120.3 (3)	C23—C22—C27	115.9 (3)
N1—C5—C7	111.1 (2)	C21—C22—C27	119.8 (3)
C4—C5—C7	128.5 (3)	C24—C23—C22	123.3 (4)
O2—C6—O1	126.2 (3)	C24—C23—H23	118.3
O2—C6—C1	120.8 (3)	C22—C23—H23	118.3
O1—C6—C1	112.9 (3)	C23—C24—C25	119.4 (4)
O4—C7—O3	125.7 (3)	C23—C24—H24	120.3
O4—C7—C5	120.2 (3)	C25—C24—H24	120.3
O3—C7—C5	114.1 (2)	C26—C25—C24	120.8 (4)
N2—C8—C9	120.7 (3)	C26—C25—H25	119.6
N2—C8—C13	111.5 (2)	C24—C25—H25	119.6
C9—C8—C13	127.8 (3)	C25—C26—C27	120.5 (4)
C8—C9—C10	117.7 (3)	C25—C26—H26	119.8
C8—C9—H9	121.2	C27—C26—H26	119.8
C10—C9—H9	121.2	N3—C27—C26	120.5 (4)
C9—C10—C11	120.6 (3)	N3—C27—C22	119.4 (3)
C9—C10—H10	119.7	C26—C27—C22	120.0 (4)
C11—C10—H10	119.7	H9A—O9—H9B	110 (4)
C12—C11—C10	118.2 (3)	H10A—O10—H10B	100 (4)
C12—C11—H11	120.9	H11A—O11—H11B	99 (3)
C10—C11—H11	120.9	H12A—O12—H12B	105 (5)
			
O3—Fe1—O1—C6	−7.7 (4)	C4—C5—C7—O3	178.1 (3)
O5—Fe1—O1—C6	−108.9 (3)	C12—N2—C8—C9	0.7 (4)
O7—Fe1—O1—C6	98.8 (3)	Fe1—N2—C8—C9	−177.7 (2)
N1—Fe1—O1—C6	−2.6 (2)	C12—N2—C8—C13	−179.4 (2)
N2—Fe1—O1—C6	174.9 (2)	Fe1—N2—C8—C13	2.2 (3)
O1—Fe1—O3—C7	5.6 (4)	N2—C8—C9—C10	−1.3 (5)
O5—Fe1—O3—C7	107.1 (2)	C13—C8—C9—C10	178.8 (3)
O7—Fe1—O3—C7	−100.8 (2)	C8—C9—C10—C11	0.8 (5)
N1—Fe1—O3—C7	0.5 (2)	C9—C10—C11—C12	0.4 (5)
N2—Fe1—O3—C7	−177.0 (2)	C8—N2—C12—C11	0.6 (4)
O1—Fe1—O5—C13	−100.7 (2)	Fe1—N2—C12—C11	179.0 (2)
O3—Fe1—O5—C13	107.1 (2)	C8—N2—C12—C14	−178.3 (2)
O7—Fe1—O5—C13	4.1 (4)	Fe1—N2—C12—C14	0.1 (3)
N1—Fe1—O5—C13	−176.9 (2)	C10—C11—C12—N2	−1.1 (4)
N2—Fe1—O5—C13	1.8 (2)	C10—C11—C12—C14	177.5 (3)
O1—Fe1—O7—C14	101.3 (3)	Fe1—O5—C13—O6	179.0 (3)
O3—Fe1—O7—C14	−105.9 (2)	Fe1—O5—C13—C8	−1.2 (3)
O5—Fe1—O7—C14	−3.4 (4)	N2—C8—C13—O6	179.1 (3)
N1—Fe1—O7—C14	177.6 (2)	C9—C8—C13—O6	−1.0 (5)
N2—Fe1—O7—C14	−1.1 (2)	N2—C8—C13—O5	−0.7 (4)
O1—Fe1—N1—C1	2.2 (2)	C9—C8—C13—O5	179.2 (3)
O3—Fe1—N1—C1	179.7 (2)	Fe1—O7—C14—O8	−177.2 (3)
O5—Fe1—N1—C1	91.7 (2)	Fe1—O7—C14—C12	1.5 (4)
O7—Fe1—N1—C1	−88.8 (2)	N2—C12—C14—O8	177.8 (3)
O1—Fe1—N1—C5	−179.6 (2)	C11—C12—C14—O8	−1.0 (5)
O3—Fe1—N1—C5	−2.1 (2)	N2—C12—C14—O7	−1.0 (4)
O5—Fe1—N1—C5	−90.1 (2)	C11—C12—C14—O7	−179.8 (3)
O7—Fe1—N1—C5	89.4 (2)	C27—N3—C15—C20	−1.3 (5)
O1—Fe1—N2—C12	−90.2 (2)	C27—N3—C15—C16	178.2 (3)
O3—Fe1—N2—C12	91.0 (2)	N3—C15—C16—C17	−179.0 (4)
O5—Fe1—N2—C12	179.3 (2)	C20—C15—C16—C17	0.5 (6)
O7—Fe1—N2—C12	0.5 (2)	C15—C16—C17—C18	−0.2 (6)
O1—Fe1—N2—C8	88.2 (2)	C16—C17—C18—C19	−0.9 (7)
O3—Fe1—N2—C8	−90.5 (2)	C17—C18—C19—C20	1.7 (7)
O5—Fe1—N2—C8	−2.2 (2)	N3—C15—C20—C19	179.6 (3)
O7—Fe1—N2—C8	178.9 (2)	C16—C15—C20—C19	0.2 (5)
C5—N1—C1—C2	−0.3 (4)	N3—C15—C20—C21	−0.7 (5)
Fe1—N1—C1—C2	177.9 (2)	C16—C15—C20—C21	179.9 (3)
C5—N1—C1—C6	−179.7 (3)	C18—C19—C20—C15	−1.3 (6)
Fe1—N1—C1—C6	−1.5 (3)	C18—C19—C20—C21	179.0 (4)
N1—C1—C2—C3	−0.7 (5)	C15—C20—C21—N4	−178.0 (3)
C6—C1—C2—C3	178.6 (3)	C19—C20—C21—N4	1.7 (5)
C1—C2—C3—C4	1.2 (5)	C15—C20—C21—C22	2.5 (4)
C2—C3—C4—C5	−0.8 (5)	C19—C20—C21—C22	−177.8 (3)
C1—N1—C5—C4	0.7 (4)	N4—C21—C22—C23	−1.7 (5)
Fe1—N1—C5—C4	−177.5 (2)	C20—C21—C22—C23	177.9 (3)
C1—N1—C5—C7	−178.8 (3)	N4—C21—C22—C27	178.0 (3)
Fe1—N1—C5—C7	3.1 (3)	C20—C21—C22—C27	−2.4 (4)
C3—C4—C5—N1	−0.1 (5)	C21—C22—C23—C24	−179.1 (4)
C3—C4—C5—C7	179.3 (3)	C27—C22—C23—C24	1.1 (5)
Fe1—O1—C6—O2	−176.8 (3)	C22—C23—C24—C25	−0.8 (6)
Fe1—O1—C6—C1	2.5 (4)	C23—C24—C25—C26	0.4 (7)
N1—C1—C6—O2	178.8 (3)	C24—C25—C26—C27	−0.4 (7)
C2—C1—C6—O2	−0.5 (5)	C15—N3—C27—C26	−177.3 (4)
N1—C1—C6—O1	−0.6 (4)	C15—N3—C27—C22	1.4 (5)
C2—C1—C6—O1	−179.9 (3)	C25—C26—C27—N3	179.5 (4)
Fe1—O3—C7—O4	−177.6 (3)	C25—C26—C27—C22	0.8 (6)
Fe1—O3—C7—C5	0.9 (4)	C23—C22—C27—N3	−179.8 (3)
N1—C5—C7—O4	176.0 (3)	C21—C22—C27—N3	0.5 (5)
C4—C5—C7—O4	−3.4 (5)	C23—C22—C27—C26	−1.1 (5)
N1—C5—C7—O3	−2.5 (4)	C21—C22—C27—C26	179.2 (3)

### Hydrogen-bond geometry (Å, º)

**Table:**

*D*—H···*A*	*D*—H	H···*A*	*D*···*A*	*D*—H···*A*
N3—H3*A*···O3	0.86	2.42	3.038 (4)	130
N3—H3*A*···O11	0.86	2.30	3.008 (4)	139
N4—H4*A*···O12^i^	0.86	2.03	2.822 (5)	152
N4—H4*B*···O5^ii^	0.86	2.39	3.115 (4)	142
O9—H9*A*···O6	0.93 (4)	1.81 (5)	2.726 (4)	165 (5)
O9—H9*B*···O11^iii^	0.92 (2)	1.85 (2)	2.766 (4)	170 (5)
O10—H10*A*···O9^iv^	0.97 (5)	1.81 (5)	2.750 (5)	164 (5)
O10—H10*B*···O1	0.97 (5)	1.90 (5)	2.859 (5)	176 (11)
O11—H11*A*···O4	0.95 (4)	1.78 (4)	2.715 (4)	165 (3)
O11—H11*B*···O8^v^	0.93 (4)	1.97 (2)	2.865 (4)	163 (4)
O12—H12*A*···O10^vi^	0.96 (6)	1.96 (7)	2.827 (5)	149 (7)
O12—H12*B*···O8	0.95 (11)	2.06 (8)	2.874 (4)	142 (10)
C4—H4···O6^vii^	0.93	2.41	3.334 (4)	171
C9—H9···O4^viii^	0.93	2.33	3.257 (4)	171
C16—H16···O12^v^	0.93	2.59	3.426 (6)	150
C17—H17···O2^ix^	0.93	2.54	3.329 (5)	143

Symmetry codes: (i) −*x*, −*y*+2, −*z*; (ii) *x*−1/2, −*y*+3/2, *z*−1/2; (iii) *x*+1, *y*, *z*; (iv) −*x*+3/2, *y*+1/2, −*z*+1/2; (v) −*x*+1/2, *y*−1/2, −*z*+1/2; (vi) *x*−1, *y*, *z*; (vii) *x*−1/2, −*y*+3/2, *z*+1/2; (viii) *x*+1/2, −*y*+3/2, *z*−1/2; (ix) −*x*+3/2, *y*−1/2, −*z*+1/2.
